# Predictors of Outcomes After Coronary Artery Bypass Grafting: The Effect of Concomitant Mitral Repair

**DOI:** 10.7759/cureus.37561

**Published:** 2023-04-14

**Authors:** Yasir O Marghalani, Jamilah AlٍRahimi, Osama K Baeshen, Abdulrahman M Alhaddad, Anas R Alserihi, Abdulaziz K Aldahlawi, Luis Z Acosta, Amir Abushouk, Fatima Ahmed, Mohammed Ahmed, Yasir M Ismail, Ayman H Elsheikh, Ali Haneef

**Affiliations:** 1 College of Medicine, King Saud Bin Abdulaziz University for Health Sciences, Jeddah, SAU; 2 College of Medicine, King Abdullah International Medical Research Center, Jeddah, SAU; 3 Cardiology, King Abdulaziz Medical City, Ministry of National Guard Health Affairs, Jeddah, SAU; 4 Cardiology, King Abdullah International Medical Research Center, Jeddah, SAU; 5 Medicine, King Saud Bin Abdulaziz University, Jeddah, SAU; 6 Cardiothoracic Surgery, King Abdullah International Medical Research Center, Jeddah, SAU; 7 Cardiothoracic Surgery, Ministry of the National Guard Health Affairs, Jeddah, SAU; 8 Cardiothoracic Surgery, King Saud Bin Abdulaziz University for Health Sciences, Jeddah, SAU; 9 Medicine, King Abdullah International Medical Research Center, Jeddah, SAU; 10 Medicine, Ministry of the National Guard Health Affairs, Jeddah, SAU; 11 Medicine, King Saud Bin Abdulaziz University for Health Sciences, Jeddah, SAU; 12 Emergency Medicine, King Abdullah International Medical Research Center, Jeddah, SAU; 13 Emergency Medicine, Ministry of the National Guard Health Affairs, Jeddah, SAU; 14 Emergency Medicine, King Saud Bin Abdulaziz University for Health Sciences, Jeddah, SAU; 15 Cardiology, Lister Hospital, Stevenage, GBR

**Keywords:** low cardiac output, myocardial infarction, lv ejection fraction (lvef), primary percutaneous coronary intervention (pci), cerebrovascular accident (stroke), chronic kidney disease (ckd), new york heart association (nyha), mitral repair, ischemic mitral regurgitation, coronary artery bypass grafting

## Abstract

Background

Ischemic mitral regurgitation (IMR) or functional MR intensity with or without repair increases the risk of coronary artery bypass grafting (CABG), and if the contaminant is undertaken, it doubles the risk of the surgery. This study aimed to characterize patients with concomitant CABG and mitral valve repair (MVR) and assess the surgical and long-term outcomes.

Methods

We conducted a cohort study from 2014 to 2020 on 364 patients who underwent CABG. A total of 364 patients were enrolled and divided into two groups. Group I (n= 349) included patients with isolated CABG, and Group II included patients who underwent CABG with concomitant mitral valve repair (MVR) (n= 15).

Results

Regarding preoperative presentation, most patients were male: 289 (79.40%), hypertensive 306 (84.07%), diabetic 281 (77.20%), dyslipidemic 246 (67.58%), presenting with NYHA classes III-IV: 200 (54.95%), and upon angiography, found to have the three-vessel disease: 265 (73%). Regarding their age mean± SD and Log EuroSCORE median (Q1-Q3), they had a mean age of 60.94± 10.60 years and a median score of 1.87 (1.13-3.19). The most prevalent postoperative complications were low cardiac output 75 (20.66%), acute kidney injury (AKI) 63 (17.45%), respiratory complications 55 (15.32%), and atrial fibrillation (AF) 55 (15.15%). Regarding long-term outcomes, most patients reported class I NYHA 271 (83.13%) and an echocardiographic decrease in MR severity. Patients with a CABG + MVR were significantly younger (53.93± 15.02 vs. 61.24± 10.29 years; P= 0.009), had a lower ejection fraction (33.6 [25-50] vs. 50 [43-55] %; p= 0.032), and had a higher prevalence of LV dilation (32 [9.17%]). EuroSCORE was significantly higher in patients with mitral repair (3.59 [1.54-8.63] vs. 1.78 (1.13-3.11); P= 0.022). The mortality percentage was higher with MVR but did not attain statistical significance. Intraoperative CPB and ischemic durations were longer in the CABG + MVR group. Furthermore, neurological complications were higher in patients with mitral repair (4 (28.57%) vs. 30 (8.65%), P= 0.012). The study’s follow-up duration median was 24 (9-36) months. The composite endpoint occurred more frequently in older patients (HR: 1.05 [95% CI: 1.02-1.09]; 0.001), patients with low ejection fraction (HR: 0.96 [95% CI: 0.93-0.99]; P= 0.006) and in patients with preoperative myocardial infarction (MI) (HR: 2.3 [95%: 1.14- 4.68]; P= 0.021).

Conclusion

Most IMR patients benefited from CABG and CABG + MVR, as evident by NYHA class and echocardiographic follow-up. CABG + MVR had a higher Log EuroSCORE risk with increased intraoperative cardiopulmonary bypass (CPB) and ischemic durations, which may have played a role in increasing the incidence of postoperative neurological complications. On follow-up, no differences were reported between the two groups. However, age, ejection fraction, and a history of preoperative MI were identified as factors affecting the composite endpoint.

## Introduction

Coronary artery bypass grafting (CABG) is the most common cardiac surgical procedure performed worldwide [1]. Several risk factors, either patient- or procedure-related, directly impact the outcomes of CABG surgery [2]. One of the important independent risk factors for increased morbidity and mortality after CABG is ischemic mitral regurgitation (IMR) [3]. Mitral valve repair combined with CABG is the recommended treatment for severe IMR; however, the management of moderate IMR is still controversial [4]. Surgical or transcatheter treatment of IMR improves survival compared to optimized medical therapy [5]. Combined CABG and mitral valve repair in treating severe IMR improves the severity of MR as compared to CABG alone [6].

There is a debate about whether to perform CABG alone or CABG combined with mitral valve repair surgery (MVR) for managing IMR; however, the most recent ESC/EACTS guidelines recommend that surgery is more likely to be considered if myocardial viability is present, mic and dilated left ventricle (LV) in the presence of normal valve leaflets, CABG alone will reverse the IMR through improved regional wall motion abnormalities, papillary muscle function, and stimulation of reverse LV remodeling without exposing the patient to increased operative risk by an additional surgical procedure [9]. Not all approaches fit all patients, and IMR is not the sole factor affecting outcomes after CABG. Thus, this study aims to assess the preoperative characteristics, intraoperative variables, postoperative events, and long-term outcomes of IMR patients undergoing either isolated CABG or CABG with MVR. 

## Materials and methods

Design and patients

We conducted a cross-sectional study on patients who underwent CABG from 2014 to 2020 at the King Faisal Cardiac Center, Jeddah, Saudi Arabia, a tertiary care center. Using convenience sampling, the study included adult patients who had CABG with or without mitral valve repair (MVR) for IMR based on an echocardiographic evaluation by the consultant cardiologist at the time. Patients who had mitral valve replacement or other concomitant cardiac procedures were excluded. Additionally, we excluded patients who had structural mitral valve disease.

Patients were divided into two groups according to concomitant mitral valve repair. Group I included patients with CABG alone (n= 349), and Group II included concomitant CABG with MVR (n= 15).

Data collection for this study was approved by the Institutional Review Board (IRB) at the King Abdullah International Medical Research Center, Jeddah, Saudi Arabia. The IRB waived the need for the patient’s consent because of the retrospective design.

Data and outcomes

We described the preoperative demographics (age, gender, and body mass index) and comorbidities [diabetes mellitus, hypertension, dyslipidemia, previous cerebrovascular accident (CVA), chronic kidney disease (CKD), history of previous myocardial infarction (MI), and previous percutaneous coronary intervention (PCI)] in all patients and compared them between the study groups. Data on left ventricular ejection fraction (EF), left ventricular dilatation, and dyskinesia was retrieved from the latest preoperative echocardiography. Angiographic data included the associated left-main coronary artery disease, three-vessel disease, or two-vessel disease. Risk stratification was performed using the log EuroSCORE [10].

Intraoperative data included cardiopulmonary bypass (CPB) and ischemic times, left internal mammary artery (LIMA) use, and the number of grafts. Postoperative outcomes were compared between both groups. Hospital outcomes were postoperative drainage in the first 24 hours after placement, low cardiac output (LCO), re-exploration for bleeding, perioperative MI (PMI), acute kidney injury (AKI), hemodialysis, neurological complications, atrial fibrillation, prolonged ventilation, the duration of hospital stay, and mortality.

Patients’ follow-ups were retrieved from the medical records. The follow-up outcomes that reflected a relatively poorer response to treatment, such as mortality, recurrence, or persistence of moderate to severe mitral regurgitation, the need for another mitral valve intervention, or repeat coronary revascularization, were grouped as the composite endpoint.

Definitions

Postoperative acute kidney injury (AKI) was defined as an increase in serum creatinine 1.5 times more than the preoperative value or the initiation of postoperative hemodialysis [11]. Neurological complications included stroke and transient ischemic attacks. Stroke was defined as the persistence of neurological impairment for more than 24 hours with radiological evidence of an ischemic or hemorrhagic insult [12]. The recent universal definition of MI is based on a rise and/or fall of cardiac biomarkers (preferably troponin) in the setting of myocardial ischemia: cardiac symptoms, ECG changes, or imaging findings. Studies using serial troponin measurements demonstrate that most PMIs start within 24 to 48 hours of surgery during the greatest postoperative stress or new regional wall motion abnormalities diagnosed with echocardiography [13]. Low cardiac output was diagnosed in the patients who required maximum inotropic support or mechanical circulatory support [14]. Prolonged ventilation was defined as postoperative mechanical ventilation lasting >24 hours. 

Techniques

The surgical procedures were all performed through a midline sternotomy using standard cardiopulmonary bypass with intermittent antegrade cardioplegia. Mitral valves were inspected to confirm the preoperative echocardiographic absence of structural pathologies. In patients undergoing MVR, bicaval cannulation was used, and mitral repair was performed after the distal anastomosis. Ring annuloplasty was the procedure of choice in all patients for MVR, and it was used according to the anterior leaflet length and inter-commissural distance. The goal of repair was to achieve a coaptation depth of 8 mm and no more than mild residual MR. The left internal mammary artery was the conduit of choice in most patients except in those undergoing emergency CABG. 

Statistical analysis

Data were presented as mean and standard deviation or median and interquartile range for quantitative data types and numbers (%) for qualitative data types. A comparison of continuous data was performed using the t-test or Mann-Whitney test. Categorical data were compared with the Chi-squared or Fisher exact test. Event-free survival was plotted using the Kaplan-Meier curve. A log-rank test was used for time-to-event data comparison. Multivariable Cox regression was performed for the composite endpoint. Model selection was performed with a stepwise forward selection method. Variables with a p-value <0.05 were retained in the final model. Variables included in the model were tested for collinearity using the variance inflation factor (VIF), and all variables included had a VIF< 1.5. Model performance was tested with Harrell’s C and proportional hazard assumptions with Schoenfeld residuals. StataCorp. 2021. Stata Statistical Software: Release 17. College Station, TX: StataCorp LLC. was used for analysis.

## Results

Preoperative and operative characteristics

The study included 364 patients who underwent CABG. Three hundred and forty-nine patients underwent isolated CABG, while 15 had CABG + MVR for IMR. The majority of patients were male: 289 (79.40%), hypertensive 306 (84.07%), diabetic 281 (77.20%), and dyslipidemic 246 (67.58%). The mean age was 60.94 years ± SD of 10.60 years. Moreover, the BMI mean± SD was 28.72± 4.95 kg/m2. Furthermore, 134 (36.81%) were smokers, 45 (12.36%) had chronic kidney disease (CKD), and 29 (7.97%) had a history of cerebrovascular accidents (CVA).

Regarding the angiographic findings of the patients preoperatively, the majority presented with three-vessel disease 265 (73%), while 71 (19.56%) had the two-vessel disease. Moreover, the left-main disease was present in 104 (28.57%) patients.

**Figure 1:**
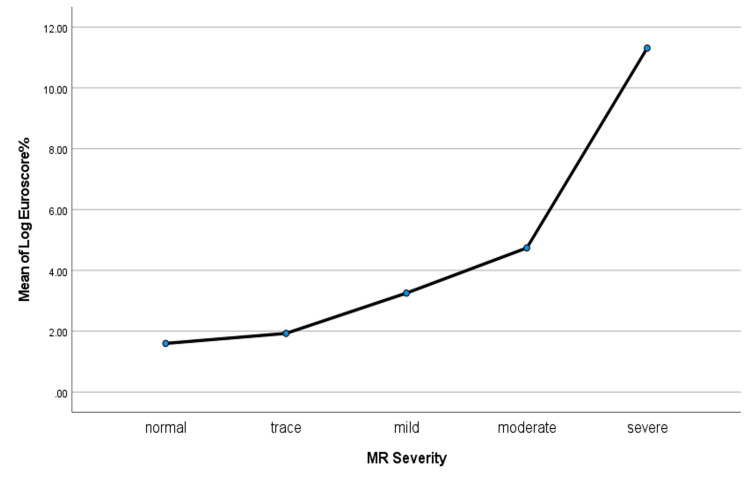
The relationship between EuroScore and mitral regurgitation grade (MR)

Regarding echocardiographic evaluation, the median ejection fraction (EF) was 50% (43-55) in the isolated CABG group and 33.6 (25-50) in the CABG + MVR group. Moreover, preoperatively, the reported severities of MR were normal, trace, mild, moderate, and severe in three (0.82%), 61 (16.76%), 258 (70.88%), 32 (8.79%), and 10 (2.75%), respectively. Furthermore, left ventricular dilation was present in 39 patients (10.71%), where 32 (9.17%) were from the isolated CABG group, and seven (46.67%) were from the CABG + MVR group. In addition, dyskinesia was present in 25 (6.89%) of the patients. When comparing the median LOG EuroSCORE among the MR severity grades, the EuroSCORE was higher in patients with a higher IMR grade, as illustrated in Figure 1.

The concomitant mitral repair patients were relatively younger (53.93± 15.02 vs. 61.24± 10.29 years; P= 0.009). Hypertension and diabetes mellitus were more prevalent in patients who had isolated CABG. Patients with CABG + MVR had a lower ejection fraction: 33.6 (25-50) vs. 50 (43-55) %; p= 0.032. There was no difference in angiographic characteristics between groups. EuroSCORE was significantly higher in patients who had mitral repair (3.59 [1.54- 8.63] vs. 1.78 [1.13- 3.11]; P= 0.022). Preoperatively, the reported severities of MR were normal, trace, mild, moderate, and severe in three (0.82%), 61 (16.76%), 258 (70.88%), 32 (8.79%), and 10 (2.75%), respectively. EuroSCORE was higher in patients with a higher IMR grade (Figure 1).

Intraoperatively, the median cardiopulmonary bypass time was longer in the CABG + MVR group: 144 minutes (109-245) vs. 100 minutes (75-127); P= 0.008. Furthermore, median ischemic time was longer as well in the CABG + MVR group: 99 minutes (73-159) vs. 58 (45-74); P=0.002. LIMA was used less frequently in patients with concomitant mitral repair compared to CABG-only patients (Table 1).

**Table 1:** Baseline characteristics and operative data of the study sample (BMI: body mass index; CABG: coronary artery bypass grafting; CPB: cardiopulmonary bypass; COPD: chronic obstructive pulmonary disease; CVA: cerebrovascular accident; CKD: chronic kidney disease; LIMA: left internal mammary artery; LV: left ventricle; MI: myocardial infarction; MVR: mitral valve repair; NYHA: New York Heart Association; PCI: percutaneous coronary intervention) *: p-value<0.05

Variables	All patients (n= 364)	CABG only (n= 349)	CABG+ MVR (n= 15)	P-value
Age (years)	60.94± 10.60	61.24± 10.29	53.93± 15.02	0.009*
Male	289 (79.40%)	278 (79.66%)	11 (73.33%)	0.553
BMI (Kg/m2)	28.72± 4.95	28.87± 4.85	25.23± 6.03	0.005*
Smoking	134 (36.81%)	127 (36.39%)	7 (46.67%)	0.419
Hypertension	306 (84.07%)	297 (85.10%)	9 (60%)	0.009*
Diabetes	281 (77.20%)	274 (78.51%)	7 (46.67%)	0.004*
Dyslipidemia	246 (67.58%)	239 (68.48%)	7 (46.67%)	0.077
COPD	18 (4.95%)	18 (5.16%)	0	>0.99
CVA	29 (7.97%)	28 (8.02%)	1 (6.67%)	>0.99
CKD	45 (12.36%)	44 (12.61%)	1 (6.67%)	0.705
Previous MI	101 (27.82%)	99 (28.45%)	2 (13.33%)	0.252
Previous PCI	86 (23.63%)	84 (24.07%)	2 (13.33%)	0.536
NYHA III- IV	200 (54.95%)	190 (54.44%)	10 (66.67%)	0.351
Ejection fraction (%)	50 (42- 55)	50 (43- 55)	33.6 (25- 50)	0.032*
LV dilatation	39 (10.71%)	32 (9.17%)	7 (46.67%)	<0.001*
Dyskinesia	25 (6.89%)	25 (7.18%)	0	0.612
Left-main disease	104 (28.57%)	101 (28.94%)	3 (20%)	0.569
3-vessels disease	265 (73%)	256 (73.56%)	9 (60%)	0.247
2-vessels disease	71 (19.56%)	68 (19.54%)	3 (20%)	>0.99
Logistic EuroSCORE	1.87 (1.13- 3.19)	1.78 (1.13- 3.11)	3.59 (1.54- 8.63)	0.022*
CPB time (min)	100 (75- 132)	100 (75- 127)	144 (109- 245)	0.008*
Ischemic time (min)	60 (45- 75)	58 (45- 74)	99 (73- 159)	0.002*
Number of grafts	2.56± 0.93	2.58± 0.89	1.87± 1.30	0.003*
LIMA use	337 (92.84%)	331 (95.11%)	6 (40%)	<0.001*

Operative outcomes

Regarding the postoperative events, the most common were LCO 75 (20.66%), AKI 63 (17.45%), respiratory complications, and AF 55 (15.15%). Regarding the length of hospital stay, the median was 13 days (10- 18). Moreover, 12 (3.31%) cases of hospital mortality were reported.

There were no differences in drainage, re-exploration for bleeding, perioperative MI, acute kidney injury (AKI), new-onset dialysis, postoperative atrial fibrillation (AF), or the length of hospital stay between the groups.

The mortality rate was higher in mitral valve patients but was not statistically significant, whereas CABG + MVR was associated with significantly higher neurological complications (Table 2).

**Table 2:** Comparison of postoperative outcomes between patients who had CABG alone vs. CABG and MVR (AF: atrial fibrillation; CABG: coronary artery bypass grafting; IABP: intra-aortic balloon pump; MI: myocardial infarction; AKI: acute kidney injury; MVR: mitral valve repair) *: p-value<0.05

Variables	All patients (n= 364)	CABG only (n= 349)	CABG+ MVR (n= 15)	P-value
IABP	52 (14.36%)	49 (14.12%)	3 (20%)	0.461
Drainage (ml/24h)	540 (370- 864)	540 (371- 854)	490 (315- 1010)	0.946
Low cardiac output	75 (20.66%)	71 (20.40%)	4 (26.67%)	0.523
Reopen for bleeding	12 (3.31%)	12 (3.45%)	0	>0.99
Perioperative MI	6 (1.65%)	6 (1.72%)	0	>0.99
AKI	63 (17.45%)	60 (17.29%)	3 (21.43%)	0.718
Hemodialysis	13 (3.60%)	12 (3.46%)	1 (7.14%)	0.407
Neurological complications	34 (9.42%)	30 (8.65%)	4 (28.57%)	0.012*
Postoperative AF	55 (15.15%)	52 (14.94%)	3 (20%)	0.484
Prolonged ventilation	30 (8.33%)	29 (8.38%)	1 (7.14%)	>0.99
Respiratory complications	55 (15.32%)	54 (15.65%)	1 (7.14%)	0.704
Hospital mortality	12 (3.31%)	10 (2.88%)	2 (13.33%)	0.083
Hospital stay (d)	13 (10- 18)	13 (10- 18)	13 (11- 14)	0.582

Follow-up

Upon follow-up, the median duration of follow-up was 24 (9-36) months, and 342 patients were available for follow-up. As shown in Table 3, by echocardiographic evaluation, 288 (92.6%) had no increase or persistence of MR severity, but the recurrence or persistence of moderate or severe MR, defined as the recurrence, refers to the worsening or returning of the mitral regurgitation abnormality after a successful CABG or repair, was found in 23 (7.4%) patients who had evidence of MR preoperatively. These 23 patients are from the group who underwent isolated CABG. Moreover, 314 (96.32%) reported NYHA classes I-II; however, there was no statistically significant difference in NYHA classes III and IV at the last follow-up between groups 11 (3.51%) vs. 1 (7.69%), P= 0.392. Furthermore, 17 (4.96%) were reported as deceased; however, no statistically significant difference was present between both groups. In addition, three (0.89%) required an additional mitral procedure, and 25 (7.4%) required revascularization. 

**Table 3:** Long-term outcomes MR: mitral regurgitation, FU; follow-up, NYHA: New York Heart Association Classification

Variable	Number (%)
Moderate to More residual MR (n=311)	
Yes	23 (7.4)
No	288 (92.6)
All-cause mortality (n= 343)	
Yes	17 (4.96)
No	326 (95.04)
Repeat Revascularization (n= 338)	
Yes	25 (7.4)
No	313 (92.60)
Repeat Mitral Procedure (n=337)	
Yes	3 (0.89)
No	334 (99.11)
FU NYHA (n=326)	
I	271 (83.13)
II	43 (13.19)
III	10 (3.07)
IV	2 (0.61)

Factors affecting the composite endpoint

**Figure 2:**
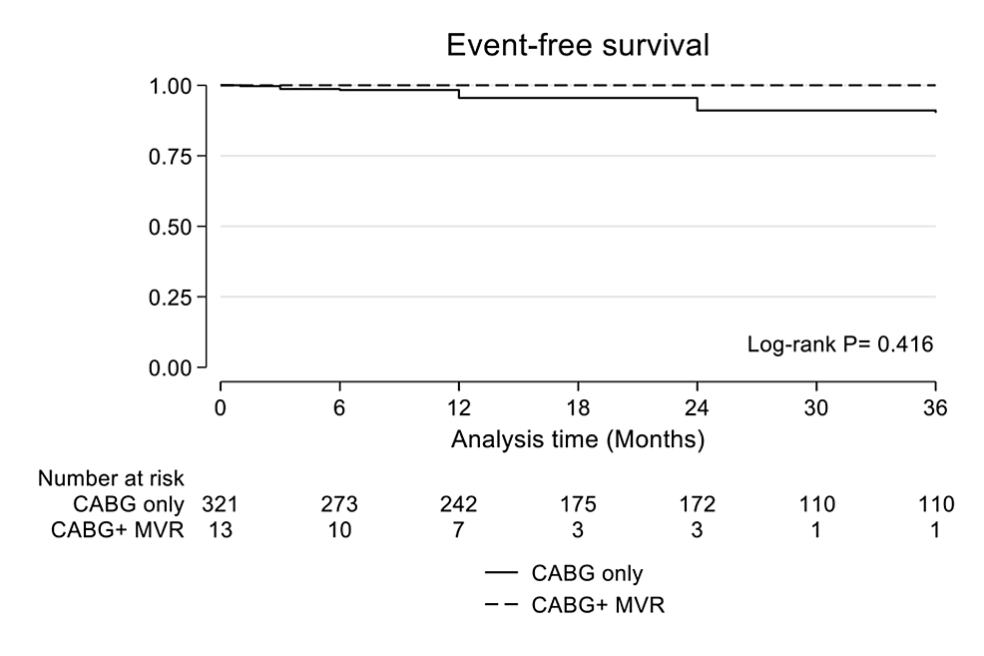
Event-free survival in patients who underwent coronary artery bypass grafting (CABG) alone vs. coronary artery bypass grafting combined with mitral valve repair (MVR)

As shown in Table 4, the composite endpoint occurred more frequently in patients of older age (HR: 1.05 [95% CI: 1.02-1.09]; 0.001), patients with low EF (HR: 0.96 [95% CI: 0.93-0.99]; P= 0.006) and in patients with preoperative myocardial infarction (HR: 2.3 [95%: 1.14-4.68]; P= 0.021). The freedom from the composite endpoint was not significantly different between groups (P= 0.416), as illustrated in Figure 2.

**Table 4:** Factors affecting the composite endpoint: (HR: hazard ratio; CI: confidence interval) *: p-value<0.05

	HR (95% CI)	P-value
Age	1.05 (1.02- 1.09)	0.001*
Ejection fraction	0.96 (0.93- 0.99)	0.006*
Myocardial infarction	2.30 (1.14- 4.68)	0.021*

## Discussion

A study done by Acker et al. concluded that IMR is associated with increased morbidity and mortality after MI [15]. Furthermore, Aklog et al. and Hickey et al. stated that there is still a debate about the optimal management of patients with moderate IMR. Patients who undergo CABG without MVR have fewer chances of having improved MR severity, negatively impacting long-term survival [16,17]. This study aimed to assess the preoperative characteristics, intraoperative variables, operative events, and long-term outcomes of IMR patients undergoing either isolated CABG or CABG with MVR. Moreover, we evaluated factors affecting the outcomes after CABG.

Regarding the baseline characteristics of the patients enrolled in this study, the majority of our patients were males, with no difference in gender distribution between CABG alone and CABG + MVR. This could be attributed to the higher prevalence of coronary artery disease in men, as concluded by Jamee et al. [18]. The most common comorbidities in our patients were hypertension, followed by diabetes mellitus, which is consistent with a nationally based study by Murray et al. about risk factors for coronary artery disease [19]. Patients with mitral valve repair were significantly younger and had a lower prevalence of hypertension and diabetes. This may be explained by the fact that patients who are considered better candidates are elected for combined CABG and MVR surgery. EF was significantly lower in CABG with MVR patients; moreover, these patients had a higher prevalence of LV dilatation. Kim and associates [20] reported comparable characteristics between patients with CABG alone versus CABG and mitral repair. However, patients with mitral repair had a significantly lower EF. The variations in preoperative variables in our series could be attributed to several factors. Patients who had repair tended to have a higher degree of MR with LV dilatation and impaired LV function. Additionally, MVR patients had a higher EuroSCORE, and we found a proportional relationship between IMR severity and EuroSCORE.

We did not find a difference in postoperative outcomes between both groups apart from higher neurological complications in combined CABG + MVR surgery. Despite being younger and having a lower prevalence of DM, increased neurological complications could be attributed to the increased complexity of undergoing concomitant CABG with MVR with prolonged cardiopulmonary bypass and ischemic times in addition to having a higher log EuroSCORE, a higher prevalence of LV dilation, and a lower median EF. Operative mortality was not significantly higher with MVR. The length of hospital stays did not differ significantly between groups. These results partially contradict Kim and colleagues’ study [20]. They reported higher neurological and cardiac complications, low cardiac output, and mortality in CABG and MVR patients.

During follow-up, recurrence or residual MR was higher in the CABG-only group. The composite endpoint did not differ significantly between groups. Our results are similar to those of other series, which found no difference in long-term outcomes between CABG with and without MVR. Bouchard and associates found no difference in EF and LV dimensions after 12 months between CABG alone and CABG and MVR [21]. Fattouch et al. [22] found that concomitant MVR was associated with improved EF, LV dimensions, and symptoms, while there was no difference in short-term survival. Similarly, Chan and colleagues [23] reported improved outcomes after MVR in their randomized clinical trial comparing CABG vs. CABG and MVR. The difference in outcomes between randomized trials and retrospective studies could be attributed to the strict inclusion criteria and the exclusion of high-risk patients.

Risk factors for the composite endpoint were age, low EF, and preoperative MI. CABG is the preferred treatment in patients with low ejection fraction and has superior outcomes compared to PCI [24]. Awan and associates [25] found that low ejection fraction is an independent risk factor for mortality in patients undergoing isolated CABG. Similar to our study, Nuru and colleagues [26] found that the risk of CABG increased in older patients. These results indicate that patient-related risk factors play a significant role in determining the long-term outcomes after CABG.

Regarding the limitations of the study, most of our patients underwent revascularization with CABG only, which can skew the data toward the latter group. In addition, patients who underwent CABG + MVR were relatively fewer in number compared to the CABG group. This issue, in addition to having a single-center study, might affect the generalizability and reproducibility of the results. Moreover, due to following a convenience sampling technique, this study is vulnerable to selection bias. Several variables may confound the outcomes and were not measured in our patients. Furthermore, the low number of postoperative events may obscure the statistically significant levels.

## Conclusions

The presence of LV dysfunction and MR in patients with CAD requiring surgical revascularization increases complexity and impacts outcomes; however, we found that concomitant CABG and MVR could increase the operative ischemic time and subsequent events, such as neurological complications. Regarding baseline characteristics, the most common comorbidities, in descending order, were hypertension, diabetes, and dyslipidemia. Moreover, preoperatively, significantly milder severities of mitral regurgitation were associated with a lower EuroSCORE. In addition, in-hospital mortality was associated with a higher mean log EuroSCORE. Regarding follow-up, there was no difference in mortality or the need for mitral valve interventions or coronary revascularization; however, recurrence or persistence of moderate or severe mitral regurgitation presented more frequently in the isolated CABG group. Moreover, age, LV dilation, and a history of MI carried a higher risk for the composite endpoint. To provide data that is more representative of the population, a multi-center study is suggested. Moreover, a prospective cohort study that includes an equally sufficient sample size in both groups could provide more valuable data.
